# Sengers syndrome: six novel *AGK* mutations in seven new families and review of the phenotypic and mutational spectrum of 29 patients

**DOI:** 10.1186/s13023-014-0119-3

**Published:** 2014-08-20

**Authors:** Alireza Haghighi, Tobias B Haack, Mehnaz Atiq, Hassan Mottaghi, Hamidreza Haghighi-Kakhki, Rani A Bashir, Uwe Ahting, René G Feichtinger, Johannes A Mayr, Agnès Rötig, Anne-Sophie Lebre, Thomas Klopstock, Andrea Dworschak, Nathan Pulido, Mahmood A Saeed, Nasrollah Saleh-Gohari, Eliska Holzerova, Patrick F Chinnery, Robert W Taylor, Holger Prokisch

**Affiliations:** Department of Genetics, Harvard Medical School, 77 Ave Louis Pasteur, Boston, MA 02115 USA; Department of Medicine and the Howard Hughes Medical Institute, Brigham and Women’s Hospital, Boston, MA USA; Institute of Human Genetics, Helmholtz Zentrum München, Neuherberg, München, Germany; Institute of Human Genetics, Technische Universität München, Trogerstrasse 22, München, 81675 Germany; Department of Pediatrics, Aga Khan University, Karachi, Pakistan; Department of Pediatrics, Imam Reza Hospital, Mashhad University of Medical Sciences, Mashhad, Iran; Faculty of Medicine, Mashhad Azad University, Mashhad, Iran; Department of Paediatrics and Neonatology, Ahmadi Hospital, Kuwait Oil Company, Al Ahmadi, Kuwait; Department of Paediatrics, Paracelsus Medical University, Salzburg, Austria; Inserm UMR 1163, Imagine Institute, Paris Descartes University, Paris, France; Department of Genetics, Hôpital Necker-Enfants Malades, Paris, France; Department of Neurology, Friedrich-Baur-Institute, Ludwig-Maximilians-Universität München, Munich, Germany; Department of Pediatric Cardiology, University Hospital, Aachen University of Technology, Aachen, Germany; Pediatric Department, Hospital Dr. Gustavo Fricke, Viña del Mar, Chile; Department of Genetics, Medical School, Kerman University of Medical Sciences, Kerman, Iran; Wellcome Trust Centre for Mitochondrial Research, Institute of Genetic Medicine, Newcastle University, Newcastle upon Tyne, UK; Wellcome Trust Centre for Mitochondrial Research, Institute of Neuroscience, Newcastle University, Newcastle upon Tyne, UK

**Keywords:** Sengers syndrome, AGK, Acylglycerol Kinase, Mutation, Genotype-Phenotype Correlation

## Abstract

**Background:**

Sengers syndrome is an autosomal recessive condition characterized by congenital cataract, hypertrophic cardiomyopathy, skeletal myopathy and lactic acidosis. Mutations in the acylglycerol kinase (*AGK*) gene have been recently described as the cause of Sengers syndrome in nine families.

**Methods:**

We investigated the clinical and molecular features of Sengers syndrome in seven new families; five families with the severe and two with the milder form.

**Results:**

Sequence analysis of *AGK* revealed compound heterozygous or homozygous predicted loss-of-function mutations in all affected individuals. A total of eight different disease alleles were identified, of which six were novel, homozygous c.523_524delAT (p.Ile175Tyrfs*2), c.424-1G > A (splice site), c.409C > T (p.Arg137*) and c.877 + 3G > T (splice site), and compound heterozygous c.871C > T (p.Gln291*) and c.1035dup (p.Ile346Tyrfs*39). All patients displayed perinatal or early-onset cardiomyopathy and cataract, clinical features pathognomonic for Sengers syndrome. Other common findings included blood lactic acidosis and tachydyspnoea while nystagmus, eosinophilia and cervical meningocele were documented in only either one or two cases. Deficiency of the adenine nucleotide translocator was found in heart and skeletal muscle biopsies from two patients associated with respiratory chain complex I deficiency. In contrast to previous findings, mitochondrial DNA content was normal in both tissues.

**Conclusion:**

We compare our findings to those in 21 previously reported *AGK* mutation-positive Sengers patients, confirming that Sengers syndrome is a clinically recognisable disorder of mitochondrial energy metabolism.

## Introduction

Sengers syndrome is a rare autosomal recessive condition. It was first described by Sengers and colleagues in 1975 with the clinical features of congenital cataract, hypertrophic cardiomyopathy, mitochondrial myopathy and lactic acidosis after exercise [1].

Two forms of this syndrome have been described, a severe neonatal form that causes infantile death and a more benign form with a longer survival into the fourth decade [2,3]. The longer surviving patients had normal developmental milestones. The cause of mortality in Sengers syndrome is heart failure as the result of hypertrophic cardiomyopathy. Van Ekeren et al. investigated 16 clinically-diagnosed patients with Sengers syndrome in order to describe the course of these two forms [2,3]. Although these authors did not observe any histopathologic distinction in muscle or heart samples between the two forms, Perry described 3 cases of neonatal Sengers with significant central nervous system involvement [4]. The cranial ultrasonography findings in the three siblings included cerebellar hypoplasia and increased echogenicity of the basal ganglia suggestive of calcification. Magnetic resonance imaging demonstrated hypoplasia of both the brainstem and inferior cerebellar vermis, impaired myelination of the cerebral hemispheres and brainstem, and cortical infarction [5]. These authors suggested that the neonatal form of Sengers syndrome should be described as a mitochondrial encephalomyopathy, because of involvement of the central nervous system. However, the genetic cause of the disease in this family has not been reported. Siriwardena et al. investigated two siblings with Sengers Syndrome and *AGK* mutations using magnetic resonance imaging and showed cortical infarction, however in a vascular pattern unlike metabolic strokes that do not respect any arterial distribution [6].

Recently, Mayr et al. investigated Caucasian Sengers patients from Germany, Italy, The Netherlands and Switzerland and, using whole exome sequencing, identified *AGK* as the disease-causing gene in Sengers syndrome [4]. *AGK* encodes a mitochondrial transmembrane enzyme, acylglycerol kinase, a multisubstrate lipid with a likely role in cardiolipin biosynthesis. Acylglycerol kinase catalyzes the formation of phosphatidic acid and lysophosphatidic acid [7] that can participate in phospholipid synthesis or act as signaling molecules regulating a number of cell processes [7–9]. Cardiolipin plays an important role in structural maintenance of mitochondria and regulating the permeability of the inner membrane. Abnormal mitochondrial morphology has been seen in conditions with cardiolipin impairment, like Sengers and Barth syndromes [10–12]. Secondary to *AGK* mutations, a deficiency of the adenine nucleotide translocator and impairment of ATP synthesis has been reported and seems to play a central role in the pathomechanism of Sengers syndrome [4,13].

We present the clinical features and molecular basis of Sengers syndrome in seven new families of different ethnic origin, documenting for the first time a molecular study of Sengers syndrome patients of non-Caucasian descent.

## Materials and methods

### Patients and families

We studied the members of seven families with Sengers syndrome in which patients were diagnosed based on the clinical features and mitochondrial histochemical and biochemical analysis. Informed written consent for clinical and molecular investigation was obtained from all family members or their legal guardians. The study was conducted in accordance with the Helsinki Declaration. The study was approved by the ethics committee of the Technische Universität München.

All members of families underwent a comprehensive clinical investigation including cardiac, metabolic, neurological, endocrinological, ophthalmological and neuromuscular assessments.

### Methods

Peripheral blood samples were collected from all family members.

The causal mutation in Case 4 (Table 1) was identified by exome sequencing as described previously [14] and subsequently confirmed by Sanger sequencing. In the other individuals, testing for DNA sequence variations in *AGK* was done by Sanger sequencing. Genomic DNA was amplified using intronic primers and the resulting PCR products were sequenced in both directions. Primer sequences and PCR conditions are available upon request.

**Table 1:** **Molecular and Clinical Findings in Patients with** ***AGK*** **Mutations**

**FEATURES**	**Sex/consan.**	**Mutation**	**Status**	**Onset of cardio-myopathy**	**Onset of cataract**	**Plasma lactic acidosis**	**Exercise intolerance**	**Motor develop. delay**	**Muscle weak.**	**Muscular hypotonia**	**Tachydys-pnea**	**OXPHOS defect**	**Other clinical features**
Case-1	Male/+	c.523_524delAT (p.Ile175Tyrfs*2)^¥^	Dead 7 m	5 m	1 m	+	Feeding difficulty	+	+	+	+		Nystagmus, floppy infant
Case-2^A^	Male/- (Fm)	c.424-1G>A (splicing defect)^¥^	Dead 10 d	Birth	Birth	+	-	-	-	-	+		-
Case-3^A^	Female/- (Fm)	c.424-1G>A (splicing defect)^¥^	Dead 4 m	Birth	Birth	+	Feeding difficulty	-	-	-	-		Eosinophilia
Case-4	Female/- (Fm)	c.409C>T (p.Arg137*)^¥^	Dead 3 m	+		+					+	I	Esotropia, IUR, feeding problems, MMA elevation in urines (14 , N>8), total and free carnitine elevation in blood, fatty infiltrations in heart biopsy
Case-5	Male/-	c.409C>T (p.Arg137*)^¥^	Dead 6 m	Birth	Birth	+	Feeding difficulty	+	+	+	+	I	Esotropia, nystagmus, floppy infant
Case-6	Male/- (Fm)	c.871C>T (p.Gln291*), c.1035dup (p.Ile346Tyrfs*39)	Alive 3 m	Birth	Birth	+	-	Mild retardation	Mild	Mild	+ RSV pneumonia		-
Case-7	Female/-	c.297+2T>C (p.Lys75Glnfs*12), c.841C>T (p.Arg281*)	Alive 10 y	Birth	Birth	-	+	+	+	+	-	I	Cervical meningocele, ragged red fibers (15%), COX-deficient fibres (1-2%)
Case-8	Male/+	c.877+3G>T (splicing defect)^¥^	Alive 15 y	+	Birth	-	+	+	-	-	-		
PC-1 [4]	Male/-	c.3G>C (p.Met1?), c.517C>T (p.Gln173*)	Alive 36 y	+	3 m	+	+	+	-	-	-		Floppy infant
PC-2 [4]	Male/- (Fm)	c.3G>C (p.Met1?), c.672C>A (p.Tyr224*)	Alive 35 y	+	3 m	On exercise	+	+	-	-	-	-	-
PC-3^B^ [4]	Male/- (Fm)	c.1131+5G>A (splicing defect)^¥^	Dead 12 y	+	18 m	-	+	-	+	+	-	I, II+III, IV, V, PDHc	-
PC-4^B^ [4]	Female/- (Fm)	c.1131+5G>A (splicing defect)^¥^	Alive 10 y	+	5 m	+	+	-	-	-	-	I, II+III, IV, V, PDHc	-
PC-5 [4]	Female/+	c.1131+5G>A (splicing defect)^¥^	Alive 41 y	+	Birth	+		+	-	-	-	V	Stroke
PC-6 [4]	Female/-	c.221+1G>A (splicing defect), c.1213C>T (p.Gln405*)	Alive 12 y	+	Birth	+	-	-	+	-	-	I, II, III, IV, very high CS	-
PC-7 [4]	Male/- (Fm)	c.412C>T (p.Arg138*), c.1137_1143del (p.Gly380Leufs*16)	Dead 11 m	+	Birth	On exercise	-	Moderate retardation	-	+	-	-	Fatty infiltrations in muscle biopsy
PC-8 [4]	Female/-	c.672C>A (p.Tyr224*), c.870del (p.Gln291Argfs*8)	Dead 10 m	+	4 m	+	-	-	-	-	-	I, II, III, IV, V, very high CS	-
PC-9 [4]	Male/-	c.101+?_222-?del (ND)^¥^	Dead 8 m	+	Birth	+	-	-	-	-	-	I, II, III, IV, V, very high CS	Axial hypotonia, upper left limbs paresis, seizures, brain ventricles dilation
PC-10 [4]	Male/-	c.306C>T (p.Tyr102*), c.841C>T (p.Arg281*)	Dead 18 d	+	Birth	+	-	-	-	-	+	I, II+III, IV, V	-
PC-11 [16]	Female/-	c.297+2T>C (p.Lys75Glnfs*12), c.1170T>A (p.Tyr390*)	Dead 18 y	+	<1 year	+			+			I, III, IV	Fatigue, failure to thrive, recurrent headaches, osteopenia and premature ovarian failure, severe mtDNA depletion in skeletal muscle
PC-12 [16]	Female/+	c.1131+1G>T (p.Ser350Glufs*19)^¥^	Dead 4 d		Birth	+				+	+	I, III, IV	Pulmonary hypertension, marked respiratory distress, thrombocytopenia, severe mtDNA depletion in skeletal muscle
PC-13 [6]	Female/+	c.979A>T (p.Lys327*)^¥^	Dead 5 m	Birth	Birth	+							Upper respiratory tract infection
PC-14 [6]	Female/+	c.979A>T (p.Lys327*)^¥^	Dead 12 d	Birth	Birth	+						I, I+III, II+III, III, IV, high CS	
PC-15 [6]	Male/+	c.979A>T (p.Lys327*)^¥^	Dead 2 d	Birth	Birth								
PC-16 [6]	Male/+	c.979A>T (p.Lys327*)^¥^	Dead 18 d	Birth	Birth								
PC-17 [6]	Male/+	c.3G>A (p.Met1?)^¥^	Dead 6 m	Birth	Birth	+		+	+				Cerebellar non-hemorrhagic stroke, ventricular , growth delayfibrillation
PC-18 [6]	Male/+	c.3G>A (p.Met1?)^¥^	Alive 2 y	2 m	Birth	-		-	-	-			Cerebellar non-hemorrhagic stroke, ventricular, modest growth delayfibrillation
PC-19 [18]	Female/+	c.424-3C>G (p.Ala142Thrfs*4)^¥^	Alive 17 y	-	Birth	-	-	-	-	-	-		Urine organic acid profile: moderately elevated lactate, 3-hydroxyisovaleric, 3-methylglutaric and 3-methylglutaconic acids
PC-20 [18]	Male/+	c.424-3C>G (p.Ala142Thrfs*4)^¥^	Alive 11 y	-	Birth	-	-	-	-	-	-		Urine organic acid profile showed moderately elevated lactate, 3-hydroxyisovaleric, 3-methylglutaric and 3-methylglutaconic acids
PC-21 [18]	Male/+	c.424-3C>G (p.Ala142Thrfs*4)^¥^	Alive 7 y	-	Birth	-	-	-	-	-	-		Urine organic acid profile showed moderately elevated lactate, 3-hydroxyisovaleric, 3-methylglutaric and 3-methylglutaconic acids
**SUMMARY**	**17 M: 12 F/13 consan.**	**20 homozygous; 19 different mutations**	**17 †; 16 † <1 y**	**25/29; 10 at birth**	**28/29 up to 18 m; 21 at birth**	**20/27**	**6/16; + 3 feeding difficulties**	**10/22**	**8/23**	**7/22**	**7/22**	**3 only I; 1 only V; 9 combined**	

+: presence of condition, −: absence of condition, unknown when empty.

I: complex I, II+III: succinate cytochrome c oxidoreductase, IV: cytochrome c oxidase, V: oligomycin-sensitive ATPase, CS: citrate synthase, OXPHOS: oxidative phosphorylation, PDHc: pyruvate dehydrogenase complex;

Fm: familial, ND: not determined;

d: day, m: month, y: year;

^A^Cases 2 and 3 are siblings;

^B^PC-3 and PC-4 are siblings;

^¥^Homozygous mutation.

The mtDNA content from 3 patient samples was measured in muscle or heart from three patients with samples available for testing according to published protocol [15]. Western blot analysis was performed from very small amount of tissue samples from 2 patients, therefore only ANT and respiratory chain complexes I and II were determined as described in [4]. Biochemical analysis of OXPHOS activity was done in 3 different research centers, where the muscle biopsies were available (case-4, 5, 7 [4]).

## Results

### Clinical investigation

Altogether, we report 8 new cases of Sengers syndrome with predicted loss-of-function mutations in all affected individuals with no hot-spot region in *AGK* gene. Six patients displayed onset of cataract at birth, one in the first month of life. Cardiomyopathy occurred at birth in 5 out of 8 cases and in two cases within the first 5 months of life. For one case, the onset of cardiomyopathy remained unclear. Five patients with severe form or Sengers syndrome died in the first year of life, last patient with severe form is alive with the age of 3 moths. Patients with milder form of disease survived the first decade of their life. Our data underline tachydyspnea and isolated Complex I deficiency as additional phenotypes of Sengers syndrome. Consistently to previous findings, blood lactic acidosis was found as a common feature for all cases with severe form of disease, most with respiratory chain deficiency. Other features like nystagmus, eosinophilia, cervical meningocele, exercise intolerance or motor developmental delay and muscle weakness differ case by case. Specific information are given in the case reports or in the overview Table 1.

#### Family-1

**Figure 1:**
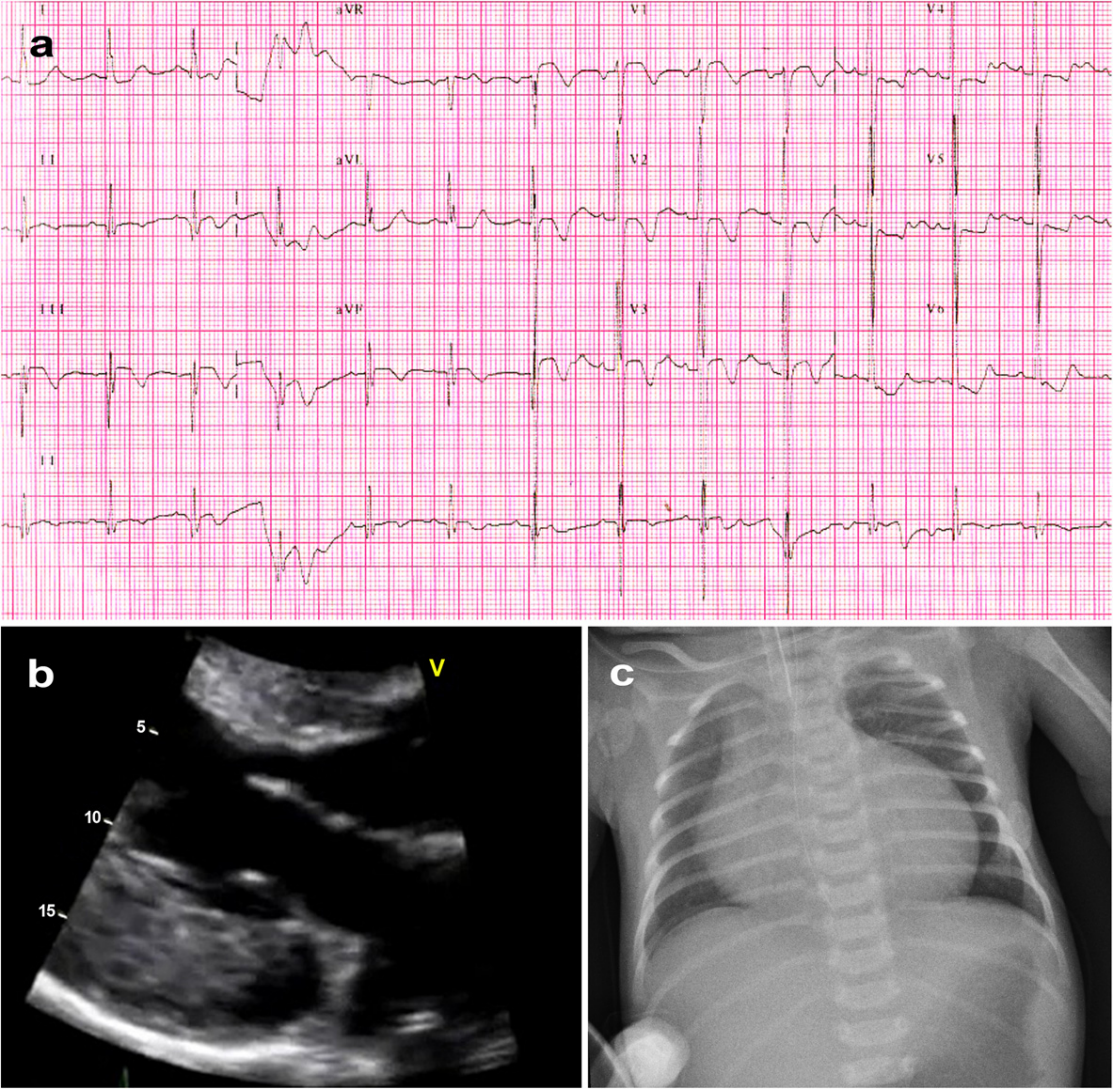
**Electrocardiogram, echocardiogram and chest X-ray of a Sengers patient. a**. ECG of Case-1 showing left ventricular hypertrophy and T wave inversion in limb and chest leads. **b**. 2D echocardiogram of in parasternal long axis projection showing severe left ventricular hypertrophy in (Case-1). **c**. Chest X-ray revealing significant cardiomegaly in (Case-3).

The first proband (Case-1, Table 1) was a five months old male child that presented with coughing and respiratory difficulties for one week, reluctance to feed and excessive crying for a few hours. There was no associated fever, vomiting or history of drug intake. He was the first born at term to healthy consanguineous parents from Pakistan. At birth, the child was diagnosed with bilateral cataract. Rubella and galactosemia were ruled out. He underwent cataract surgery uneventfully during the first month of life. He had subsequent admissions for eye examinations under anesthesia and circumcision; both admissions were without complications. The child was noticed to sweat and get tired during feeding. His physical growth was normal. Motor milestones were delayed and the child was unable to hold his neck. Family history was significant with neonatal deaths in two paternal uncles. On admission, he had supraventricular tachycardia with a heart rate of 230 bpm with congestive cardiac failure. The respiratory rate was 46/m with acidotic breathing pattern and blood pressure was 80/60 mmHg. The heart sounds were distant and there was no murmur. He had moderate hepatomegaly. His eyes were aphakic, pupils being irregular shaped, sluggishly reactive to light. He also had generalized hypotonia. Electrocardiogram was consistent with supraventricular tachycardia. This was aborted with cardioversion and a repeat electrocardiogram showed left ventricular hypertrophy and T wave inversion in limb and chest leads (Figure 1a). Arterial blood gases depicted severe metabolic acidosis (pH 6.9, pCO_2_ 32.6 mmHg, pO_2_ 98 mmHg, HCO_3_ 6 meq/L) and anion gap was 29 meq/L. Serum lactate level was 20 mmol/L (normal 0.67-2.67 mmol/L). Echocardiography was consistent with hypertrophic non-obstructive cardiomyopathy (Figure 1b) with a severe global systolic dysfunction (ejection fraction 15%). Moderate mitral and tricuspid valve regurgitation were present. The child was suspected to have Sengers syndrome. A skeletal muscle biopsy taken from gastrocnemius muscle was stained with routine stains and examined under light microscopy. Abnormal muscle fibers were found with histiocytic infiltrates. Special stains did not show ragged red fibers nor storage cells. Cytochrome *c* oxidase histochemistry and electron microscopy were not available to study abnormal mitochondria.

#### Family-2

**Figure 2:**
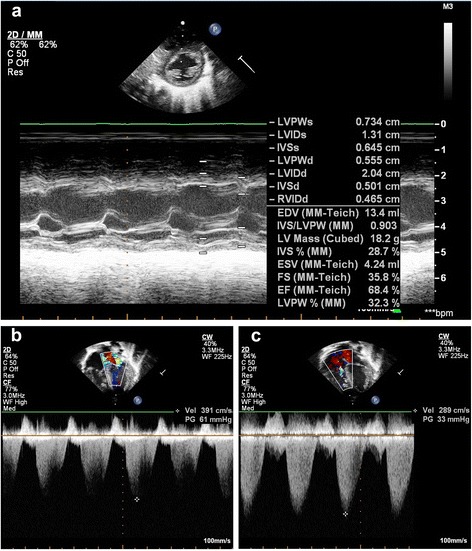
**Echocardiography of a Sengers patient. a**. M-Mode of LV in short axis showing dilated LV for age of (Case-3) at 6 days (LVEDD 2.04). **b**. moderate mitral valve regurgitation with left atrial contraction in systole. **c**. moderate tricuspid regurgitation with right atrial contraction envelope in systole.

The second proband (Case-2, Table 1) was a full term baby boy born to a 29 year old gravida-2 mother by vacuum extraction for fetal decelerations. His birth weight was 3.28 kg. Apgar score was 5, 8, 8 at 1, 5, 10 minutes, respectively. There was cord round the neck tight and oligohydramnios. He responded well to resuscitation. The antibodies for toxoplasmosis were negative. A previous sibling (Case-3, Table 1) was a term girl with bilateral congenital cataract and congenital heart disease. Her echo showed fenestrated atrial septal defect (ASD) and interventricular septal hypertrophy. Anticongestive medications were started for the neonate but she died at the age of 4 months. There was an episode of high lactate while the girl was in the neonatal intensive care unit, associated with diarrhea which self-recovered. Parents were non consanguineous, but they hailed from the same village in India. At birth, the baby was noted to be hypoperfused, tachypneic and with grade 3/6 pan-systolic murmur over the left sternal edge. Initial blood gas showed a mild metabolic acidosis, which normalized after a normal saline bolus. Chest X-ray showed gross cardiomegaly (Figure 1c). Ophthalmology consultation revealed bilateral lamellar cataract. Echocardiography (Figure 2) at 48 hours of life revealed moderate secundum ASD (5 mm), pulmonary arterial pressure 80% at the systemic level, moderate right ventricular hypertrophy and right atrial dilatation. The ejection fraction was 54%. As per the advice of the cardiologist, he was started on oral sildenafil, furosemide and dopamine. The tachypnea gradually improved and finally he could be maintained on full feeds. Ultrasound evaluation of brain and abdomen were normal. Repeat echocardiography after 72 hours showed hypertrophic left ventricle with ejection fraction 50% and improvement of pulmonary hypertension. Maintenance digoxin was started along with increasing doses of captopril. Sildenafil and dopamine were stopped and furosemide and spironolactone were continued. Basic metabolic work-up showed serum lactate of 14.5 mmol/L, which on repeat was 6 mmol/L and ammonia and blood gas levels were normal. At the age of 10 days, he was irritable, pale and desaturating. He became bradycardic. Blood gas tests showed severe metabolic acidosis and chest X-ray revealed increasing cardiomegaly. Despite the resuscitative efforts, baby expired.

#### Family-3

The proband (Case-4, Table 1) was a girl, the second child from healthy non consanguineous Indian parents. They previously lost a first child at 3 days of life probably due to infection. She was born after an uneventful pregnancy. Bilateral cataract was noticed at birth. When she was 2.5 months old, her severe hypertrophic cardiomyopathy was detected after she collapsed during cataract surgery. The girl had growth retardation (weight 3.770 g/-1.5SD, head circumference 36 cm/-1.5SD), strabismus and feeding difficulties. She presented hyperlactatemia and hyperlactaturia. Plasma glutamine, alanine, proline, and free and total carnitine levels were elevated. Methylmalonic acid was raised in urine (14 μmol/mmol creatinine, normal < 8 μmol/mmol). An isolated complex I deficiency was found in endomyocardial biopsy, whereas cultured skin fibroblasts showed normal respiratory chain activities. A heart biopsy revealed fatty infiltrations. She died at the age of 3 months from heart failure.

#### Family-4

The male patient (Case-5, Table 1) was born at term by vaginal delivery. The newborn was initially in a good condition with a normal Apgar score of 9-10-10. Later on, he started to present symptoms of heart failure with tachypnea and hepatomegaly. Echocardiography showed profound concentric hypertrophy of the left ventricle. The cavum of the left ventricle was almost completely obstructed but left ventricular outflow tract was well developed and unobstructed. Anticongestive treatment with diuretics was started, but did not improve the condition. He also developed lactic acidosis. Physical examination showed muscular hypotonia and dystrophic features. Ophthalmologic examination showed bilateral cataract that was operated later on. Muscle biopsy excluded Pompe disease, but showed low activity of mitochondrial respiratory chain complex I. In the further course, the child developed a mild form of necrotizing enterocolitis with intestinal bleeding and air collection in the portal vein. Antibiotic therapy was started while he was kept on parenteral nutrition for a couple of days that made him clinically more stable. Furthermore, he was placed on ketogenic diet, riboflavin (40 mg/d) and coenzyme Q 10 (40 mg/d). In addition, propranolol was started because of heart failure. At the age of two months, the infant was discharged from hospital on parents’ request. He passed away three months later, at the age of five months.

#### Family-5

The proband (Case-6, Table 1) was a 3 months boy infant, from Chile, who was admitted because of respiratory syncytial virus (RSV) bronchiolitis and pneumonia. He was born to healthy non-consanguineous parents. On chest radiography, his heart was enlarged. He was diagnosed with bilateral cataract since birth too. Echocardiography revealed non-obstructive hypertrophic cardiomyopathy with poor left ventricular function. His older brother died at the age of 15 months due to severe heart failure and cardiogenic shock. He also had hypertrophic cardiomyopathy, bilateral cataract and lactic acidosis. Autopsy pathology showed cytosolic granules in heart muscle and liver cells. He also had neurological developmental delay and mild hypotonia. Severe viral respiratory infection was reported in this patient as well.

#### Family-6

The patient (Case-7, Table 1) was a 10 year old girl, born to non-consanguineous healthy parents. The pregnancy was complicated by early bleeding. The heart abnormality was detected at the 19 week ultrasound scan. She was born by vaginal delivery and required minimal resuscitation. On physical exam at birth, the absence of a red reflex led to diagnosis of cataract that was operated in infancy. As an infant she was also diagnosed with a cervical meningocele, noted at birth, and underwent a surgical operation within 6 months of life. Echocardiography at the 7 days of age, revealed a hypertensive hypertrophic cardiomyopathy. She walked at 18 months of age and had significant speech and language delay with only minimal vocalization at the age of 2 years. On physical examination at 2 years of age, all growth milestones (height, weight and head circumference) were below 4^th^ centile, she had poor muscle mass and hypotonia but no overt dysmorphic features. She had normal cognitive functions and later went to normal school. She had growth hormone deficiency that was treated by growth hormone. The abnormal clinical findings at the last visit, at the age of 10 years, included exercise intolerance, fatigue, hypotonia, and stable cardiomyopathy. Muscle biopsy revealed an isolated complex I deficiency in muscle.

#### Family-7

The proband (Case-8, Table 1) was a 15 year old boy, the second child of healthy consanguineous parents. Growth retardation, cataract, muscular hypotonia and moderate motor retardation were noted in infancy by the parents. Rubella and galactosemia were ruled out. On physical examination, at 2 years of age, he had bisferiens carotid pulse, grade 2–3/6 systolic murmur, normal S1 and single S2. Echocardiography revealed left and right ventricular outflow tract obstructions, significantly increased left ventricular mass index, biventricular diastolic dysfunction and mitral valve regurgitation. Carnitine therapy was initiated for the patient but no clinical improvement was observed. His younger brother had also had cataract, cardiomyopathy and metabolic disorder and died at the age of 3 months.

### *AGK* mutation analysis

Sequencing analysis of *AGK* identified disease-causing mutation in all patients. Molecular analysis of the genomic DNA from Family-1 identified a novel homozygous 2 bp deletion (c.523_524delAT) mutation in the proband, predicting a frameshift (p.Ile175Tyrfs*2) in the translated protein. Both parents were confirmed to be heterozygous carriers.

In Family-2, sequencing of their genomic DNA revealed a novel homozygous canonical splice site mutation c.424-1G > A in the proband, affecting exon 8. Both parents and a healthy sibling were heterozygous for this mutation.

Molecular study in families 3 and 4 detected a homozygous nonsense mutation, c.409C > T, predicting a premature termination codon at position 137 of AGK, p.Arg137*. The parents, in both families, were heterozygous for this mutation.

Genetic study of Family-5 identified novel loss-of-function compound heterozygous mutations, a nonsense variant c.871C > T, (p.Gln291*), in exon 12, and a one-nucleotide insertion c.1035dup in exon 14 that is predicted to result in a frameshift (p.Ile346Tyrfs*39). The healthy parents were heterozygous for either of these two mutations.

Sequencing of the proband in Family-6, identified a heterozygous nonsense mutation c.841C > T (p.Arg281*) in combination with a heterozygous canonical splice site mutation c.297 + 2 T > C that has been shown to cause a shortened transcript with a premature termination codon (p.Lys75Glnfs*12) [16].

Molecular analysis of Family-7 revealed a homozygous mutation (c.877 + 3G > T) that is likely to affect splicing of exon 12.

**Figure 3:**
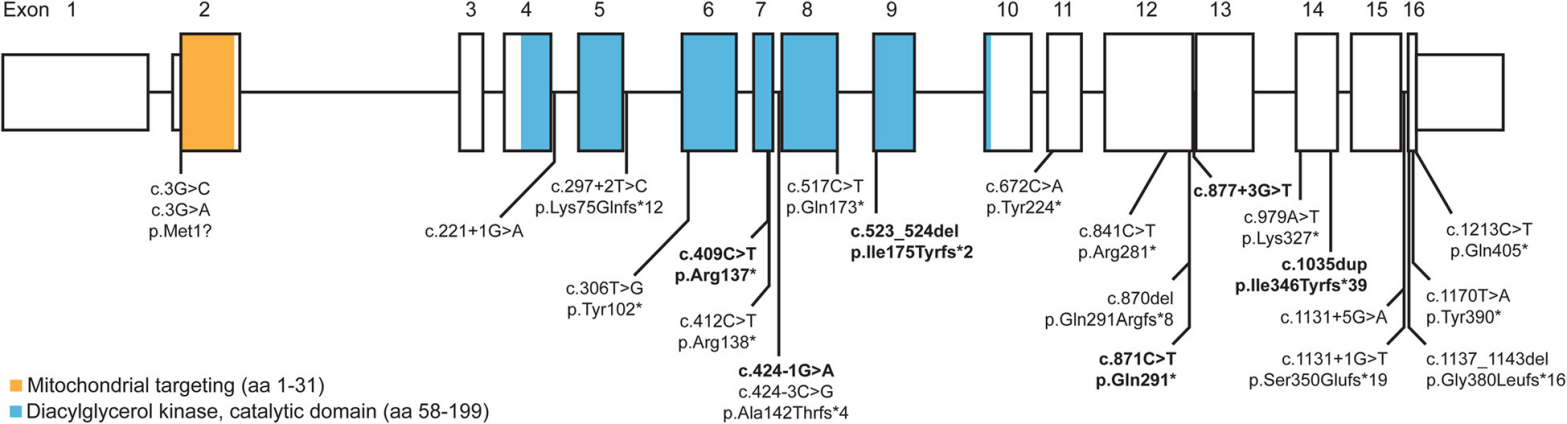
**Gene structure of** ***AGK*** **and localization of identified mutations.** Boldface type indicates newly reported mutations.

The results of genetic analysis are summarized in Table 1. Figure 3 illustrates the localization of identified mutations in the *AGK* gene.

### Immunoblotting

**Figure 4:**
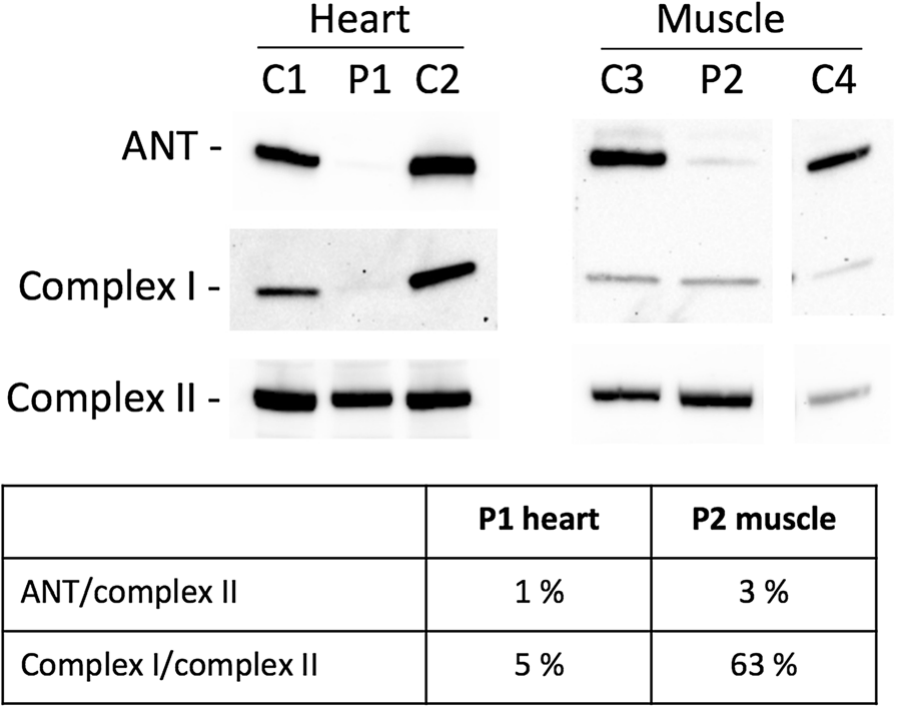
**Western blot analysis of heart and muscle samples.** The blots were decorated with antibodies (all from Mitosciences/Abcam (Eugene, OR, USA)) against adenine nucleotide translocator (ANT), subunit NDUFS4 of complex I, and subunit SDHA of complex II. Intensities were quantified by the Image J software (National Institutes of Health, Bethesda, MD) and relative intensities were calculated for the patient sample in each tissue in relation to the two controls. P1stands for PC-8 and P2 stands for Case 5 in the Table 1.

Western blot analysis was performed with the muscle biopsy sample of the patient from Family-4 and a heart biopsy sample of a previously published patient (PC-8, Table 1), diagnosed with combined respiratory chain deficiency [4]. Antibodies against the adenine nucleotide translocator revealed a clear decrease in both the heart and the muscle sample (Figure 4). Complex I was moderately decreased in muscle but severely decreased in the heart sample (Figure 4).

### Quantification of the mitochondrial DNA copy number

The relative mitochondrial DNA content in the muscle biopsy of the patient from families 4 and 6 and the heart sample of the previously published patient PC-8 was analyzed by quantitative real time PCR (qRT-PCR) [15]. A high normal value of 1750 ± 390 copies of mitochondrial DNA (mtDNA) per nuclear genome (normal range 750-1610) was found in the muscle samples from case 5 and levels within the normal range for case 7 (130% of the mean from controls). The relative mtDNA content in the heart sample was 4240 ± 730 copies of mtDNA per nuclear genome which is within a range of two controls (1042 and 5131).

## Discussion

Sengers syndrome is caused by mutations in the *AGK* gene [4], which is located on chromosome 7q34 and consists of 16 exons. To date, only three studies have investigated families with Sengers syndrome and identified different types of loss-of-function mutations in *AGK* gene, including start codon mutations (compound heterozygous), nonsense (compound heterozygous), frameshift (compound heterozygous) and splice site mutations (homozygous and compound heterozygous) [4,16]. An overview about the clinical, biochemical and genetic findings of all published and recently identified patients is summarized in Table 1. We investigated the clinical features and molecular basis of Sengers syndrome in seven new affected families. We have identified six novel predicted loss-of-function mutations in *AGK*; homozygous c.523_524delAT (p.Ile175Tyrfs*2), c.424-1G > A (splice site), c.409C > T (p.Arg137*) and c.877 + 3G > T (splice site), and compound heterozygous c.871C > T (p.Gln291*) and c.1035dup (p.Ile346Tyrfs*39). Figure 3 provides an overview of the known and newly identified mutations.

To date, less than 10 families with neonatal Sengers syndrome have been reported [4,5]. The average survival of the severe form (with onset in the first month of life), in our and previously published cases, was 4.2 months, whereas Mayr et al. reported patients with the milder form of the disease who survived through their 5^th^ decade of life (cases PC-5, PC-1 and PC-2, Table 1). Very early mortality (<5 days of life) has also been reported (cases PC-12 and PC-15, Table 1). Calvo et al. described truncating *AGK* mutations in two patients with bilateral cataract, severe myopathy and combined complex I, III and IV deficiency [16]. The first patient (PC-11, Table 1) was born to unrelated parents and harbored compound heterozygous mutations; a splice site mutation c.297 + 2 T > C (p.Lys75Glnfs*12) resulting in a shortened transcript, and a nonsense mutation c.1170 T > A (p.Tyr390*). Her other clinical features included failure to thrive, fatigue, recurrent headaches, osteopenia and premature ovarian failure. She died at the age of 18 months. The second patient (PC-12, Table 1), born to a consanguineous family, was homozygous for a splice site mutation, c.1131 + 1G > T (p.Ser350Glufs*19), that causes a shortened transcript. The infant had severe lactic acidosis (10 –17 mmol/L, normal 0.7–2.0) (John Christodoulou, personal communication) and metabolic acidosis, and died at 4 days of age due to cardiorespiratory collapse [16].

Tachydyspnoea was a feature which was observed only in severe from of the disease (Table 1), and not in any of patients with the milder form. This feature seems to be correlated with poorer prognosis and survival (average ~ 2.8 months).

The unusual features in our patients were nystagmus (Case-1 and Case-5), eosinophilia (Case-3) and cervical meningocele (Case-7). In addition, esotropia was found in two patients (Case-4 and Case-5) with the c.409C > T (p.Arg137*) mutation.

We did not identify a hot-spot region within the *AGK* gene showing clustering of mutations; these were equally distributed over the whole gene sequence. So far, all *AGK* mutations associated with Sengers syndrome are predicted to be loss-of-function variants. However, we noted that homozygous *AGK* nonsense mutations have always resulted in infantile (severe) form of the Sengers syndrome while all patients who survived the first decade harbor at least one splice site variant or a start codon mutation. In fact, two out of the three oldest patients with survival of >35 years carry the c.3G > C (p.Met1?) mutation in combination with two different stop codon mutations and the third one was homozygous for the splice mutation c.1131 + 5G > A, frequently found in patients from The Netherlands. This observation may indicate some AGK activity through residual normal splicing or alternative start codon usage[17].

Very recently, Aldahmesh et al. identified a splice site mutation in *AGK* causing isolated congenital cataract (recessive cataract 38) in a multiplex consanguineous family from Saudi Arabia, thereby extending the mild clinical spectrum associated with *AGK* mutations to a non-syndromic form. Urine organic acid profile showed moderately elevated lactate, 3-hydroxyisovaleric, 3-methylglutaric and 3-methylglutaconic acids (Fowzan Alkuraya, personal communication) and interestingly, cardiology evaluation of the affected family members revealed normal results (Table 1). The identified mutation, c.424-3C > G, is predicted to lead to aberrant splicing and predicted premature truncation, p.Ala142Thrfs*4 [18]. It can be speculated therefore that a small proportion of the normally spliced transcript can still be formed.

Although the role of *AGK* mutations in cataractogenesis is unclear, some authors have raised the hypothesis that an impairment of lenticular lipid composition may be of pathophysiological significance in the etiology [18,19]. In our and previously published cases with the severe form of Sengers syndrome, cataract was present at birth or detected shortly thereafter, whereas in milder form, it can remain undiagnosed for several months (18 months of age in PC-3, Table 1).

Several patients (Case-7, Case-8, PC-3 and PC-18, Table 1) with a milder form of Sengers syndrome did not develop lactic acidosis, whereas in the severe form, none of the patients had normal levels of lactic acid. Siriwardena et al. reported a Sengers patient with the start codon mutation, p.Met1?, who interestingly, at the age of 3 years, did not have skeletal myopathy. He was able to run and play without any limitation and his creatine phosphokinase levels were also normal. His skeletal muscles biopsy, at the age of 4 months, did not show presence of COX negative fibers [6]. However, his sibling, obviously with the same *AGK* mutation, was affected with the severe form of the diseases and died at the age of 6 months. These observations suggest that myopathy can develop independent from lactic acidosis and that the absence of lactic acidosis favors a better prognosis with longer-term survival, even in patients with the same *AGK* mutations. This observation resembles patients with Barth syndrome, which is known to show clinical variability found in siblings with identical *TAZ* genotype [20]. Remarkably, these two mitochondrial disorders also seem to result in a decrease of complex I, which has been demonstrated by western blot analysis in Barth syndrome [21] and shown here in a heart and to milder extent a skeletal muscle sample (Figure 4).

Based on the finding of severe mtDNA depletion in skeletal muscle in two patients reported by Calvo and colleagues [16], Sengers syndrome is now also called cardiomyopathic mitochondrial DNA depletion syndrome-10 (OMIM 212350). In contrast to this published study, we did not find any indication for mtDNA depletion in muscle or heart from three patients with samples available for testing. Moreover, in the original publication of the clinical syndrome by Sengers and colleagues [1], the biochemical analysis of mitochondrial enzyme complex activities revealed normal results, which is incompatible with a severe loss of mtDNA copy number [1,6]. To date, only nine patients with *AGK* mutations have been reported to have a combined respiratory chain deficiency indicating that mtDNA depletion is not a common feature of Sengers syndrome.

Based on our findings, we can describe two distinct forms of Sengers syndrome, an infantile or severe form and a mild form. The infantile form of Sengers syndrome is caused by homozygous *AGK* nonsense mutations and is characterized by early onset (within the first few months of life) cardiomyopathy and lactic acidosis that causes death in infancy. Some patients who carry at least one AGK splice site variant or a start codon mutation develop a milder form of Sengers syndrome and have a markedly better prognosis. These patients usually develop cardiomyopathy in later stages. They can survive even through their 5^th^ decade of life and in the extreme case, they may only develop cataract.

Currently, no treatment is available for this fatal condition; therefore it is important to identify carriers in at-risk families in order to provide genetic counseling and prenatal diagnosis.

In summary, we have identified six novel *AGK* mutations causing Sengers syndrome. Our findings, along with the other studies, indicate clinical heterogeneity in patients harboring *AGK* mutations. However, interestingly, congenital or early-onset cataract is the common phenotypic finding in all patients, suggesting a role for *AGK* in the homeostasis of the human eye lens. We also suggest that infants with cataract, even in the absence of cardiomyopathy, should be screened for Sengers syndrome or *AGK* mutations. This study verifies the causative role of *AGK* in Sengers syndrome and expands the genotype-phenotype correlations of mutations in this gene. Our results have important applications in genetic counseling and prenatal diagnosis.

## Abbreviations

AGK: Acylglycerol kinase
ANT: Adenine nucleotide translocator
ASD: Atrial septal defect
RSV: Respiratory syncytial virus
mtDNA: Mitochondrial DNA
